# β-D-Galactose-Functionalized Pillar[5]arene With Interesting Planar-Chirality for Constructing Chiral Nanoparticles

**DOI:** 10.3389/fchem.2019.00743

**Published:** 2019-11-14

**Authors:** Guangping Sun, Liangtao Pu, Srikala Pangannaya, Tangxin Xiao, Xiao-Yu Hu, Juli Jiang, Leyong Wang

**Affiliations:** ^1^Key Laboratory of Mesoscopic Chemistry of Ministry of Education, Jiangsu Key Laboratory of Advanced Organic Materials, School of Chemistry and Chemical Engineering, Nanjing University, Nanjing, China; ^2^College of Material Science and Technology, Nanjing University of Aeronautics and Astronautics, Nanjing, China; ^3^School of Petrochemical Engineering, Changzhou University, Changzhou, China; ^4^State Key Laboratory of Pollution Control and Resource Reuse, School of Environment, Nanjing University, Nanjing, China

**Keywords:** supramolecular macrocycles, β-D-galactose-functionalized pillar[5]arenes, planar chirality, self-assembly, nanoparticles

## Abstract

Planar-chiral pillar[5]arenes bearing β-D-galactose substituents on both rims have been successfully synthesized and effectively separated by silica gel chromatography with a high yield. The obtained (*S*_*p*_)- and (*R*_*p*_)-β-D-galactose functionalized pillar[5]arenes [(*S*_*p*-*D*_)-**GP5** and (*R*_*p*-*D*_)-**GP5**] exhibit the *S*_*p*_ and *R*_*p*_ planar chirality. Furthermore, (*S*_*p*-*D*_)-**GP5** and (*R*_*p*-*D*_)-**GP5** can not racemize according to dynamic ^1^H NMR and CD spectra. Notably, **GP5** is able to capture a guest molecule (**DNS-CPT**) to form a host-guest supramolecular amphiphile, which can further self-assemble into chiral nanoparticles with the *S*_*p*_ and *R*_*p*_ planar chirality of (*S*_*p*-*D*_)-**GP5** and (*R*_*p*-*D*_)-**GP5** still being retained, suggesting **GP5** could be as reliable chiral sources to transfer the *S*_*p*_ and *R*_*p*_ planar chirality.

## Introduction

Supramolecular macrocycles, such as cyclodextrins, cucurbiturils, and calixarenes, have played a very important role in supramolecular chemistry (Moghaddam et al., 2011; Zhang and Wang, 2011; Jie et al., 2015; Choi et al., 2017). Compared with these traditional macrocycles, pillar[n]arenes have attracted more attention due to their unique planar chirality (Ogoshi et al., 2011b). The planar chirality of pillar[n]arenes is very useful for chiral molecular recognition, chirality switches, and catalysis because of the outstanding host-guest properties of pillar[n]arenes to capture different guest molecules (Yao et al., 2017; Lee et al., 2018; Park et al., 2019).

As many literatures have presented (Ogoshi et al., 2011a, 2012, 2013a,b, 2016; Kitajima et al., 2014), the planar chirality of pillar[n]arenes is mainly caused by the substitution position of the alkoxy moieties. Ogoshi et al. (2012) and Kitajima et al. (2014) found that all of the synthesized pillar[5]arenes are racemic mixtures and racemization takes place by rotation of units. These racemic mixtures could be divided into eight conformers including diastereomeric conformers: (*Sp, Sp, Sp, Sp, Sp*), (*Rp, Sp, Sp, Sp, Sp*), (*Rp, Rp, Sp, Sp, Sp*), (*Rp, Sp, Rp, Sp, Sp*) and their antipodal enantiomers: (*Rp, Rp, Rp, Rp, Rp*), (*Sp, Rp, Rp, Rp, Rp*), (*Sp, Sp, Rp, Rp, Rp*), (*Sp, Rp, Sp, Rp, Rp*). In order to isolate the different pillar[5]arene enantiomers, they have functionalized pillar[5]arene with 10 bulky cyclohexylmethyl groups at both rims to inhibit the rotation of the units (Ogoshi et al., 2011a). Then, two special enantiomers [(*Sp, Sp, Sp, Sp, Sp*) and (*Rp, Rp, Rp, Rp, Rp*)] were successfully separated by chiral high performance liquid chromatography (HPLC). The circular dichroism (CD) spectra of the two enantiomers were clearly defined with a complete mirror image, which was defined as (*S*_*p*_)- and (*R*_*p*_)-pillar[5]arenes, respectively. Simultaneously, Strutt et al. (2012, 2014) reported the separation of pillararene-based enantiomers by introducing one π-conjugated unit, which expressed good and selective encapsulation of neutral and positively charged electron poor aromatic guests. Moreover, some other researches (Yao et al., 2017; Lee et al., 2018; Park et al., 2019) about planar chirality of pillar[5]arenes have been performed to achieve chiral inversion, chiral transfer and so on. Besides the above mentioned pillar[n]arene derivatives, β-D-galactose-functionalized pillar[5]arene (**GP5**), as a new-type of sugar modified supramolecular amphiphile, has been widely used in biologically relevant fields for the construction of antibacterial and targeted drug delivery systems (Nierengarten et al., 2013; Yu et al., 2013; Liu et al., 2017; Wu et al., 2017). However, all the results above never revealed the planar chirality of **GP5**, and there was no report about the investigation of (*S*_*p*_)- and (*R*_*p*_)-β-D-galactose-functionalized pillar[5]arene [(*S*_*p*-*D*_)-**GP5** and (*R*_*p*-*D*_)-**GP5**]. In our previous work (Liu et al., 2017), we have obtained a similar β-D-galactose-based water-soluble pillar[5]arene (**GalP5**), which showed no planar chirality, because **GalP5** possessed one methylene group at the position of β-D-galactose, resulting in the disappearance of planar chirality induced in the progress of functionalized pillar[5]arenes. Herein, we have successfully designed a new β-D-galactose functionalized pillar[5]arene without the presence of methylene group connected with β-D-galactose, and first achieved the separation of diastereoisomers possessing planar chirality by silica gel chromatography to obtain (*S*_*p*-*D*_)-**GP5** and (*R*_*p*-*D*_)-**GP5** with a high yield. Their rotational and planar chiral properties were investigated by NMR, UV-Vis and CD measurements, respectively.

## Results and Discussion

### Planar Chirality of GP5

**Figure 1:**
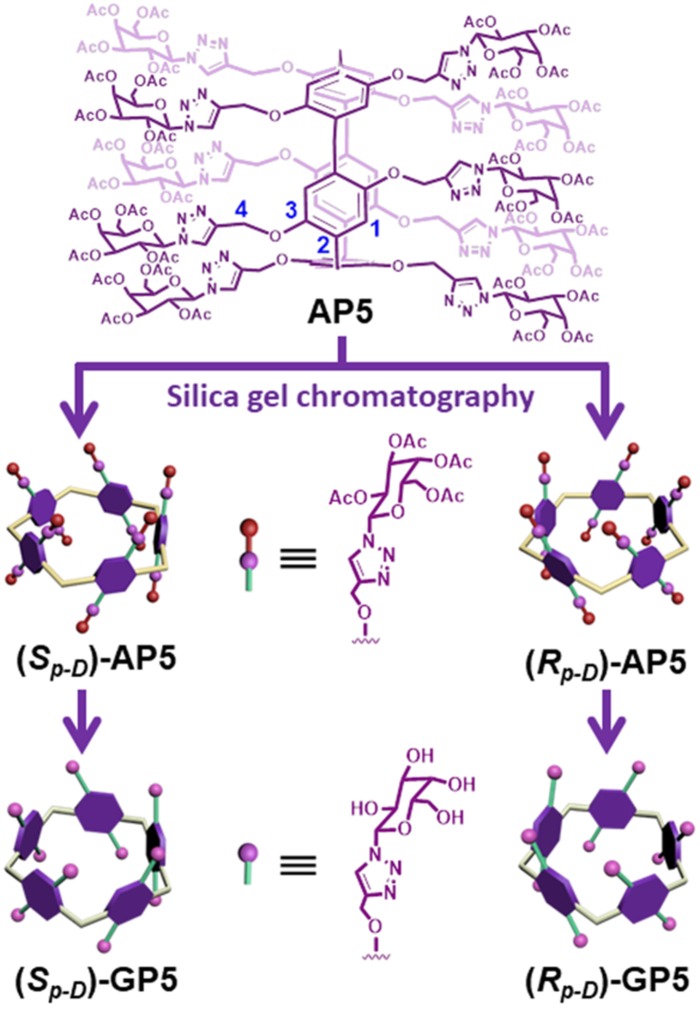
Schematic structure illustration of **AP5** and **GP5**.

The synthesis of **GP5** relies on the copper-catalyzed alkyne-azide cycloaddition (CuAAC) reaction, which was used to introduce the bulky β-D-acetylgalactose moieties on both rims of the pillar[5]arene building block. In this way, it can effectively inhibit the rotation of the units and thus achieve the separation of the (*S*_*p*_)- and (*R*_*p*_)-β-D-acetyl-galactose pillar[5]arene [(*S*_*p*-*D*_)-**AP5** and (*R*_*p*-*D*_)-**AP5**] (Figures 1, **9**). From the ^1^H NMR spectrum of **AP5**, we can clearly find that the resonances of the aromatic protons (H_1_) show two single peaks, identifying the existence of (*S*_*p*-*D*_)-**AP5** and (*R*_*p*-*D*_)-**AP5** (Figure S19). In order to further investigate the planar chirality of **AP5**, (*S*_*p*-*D*_)-**AP5** and (*R*_*p*-*D*_)-**AP5** were successfully obtained by silica gel chromatography with DCM/MeOH = 40:1 as fluent solvent. As shown in Figure S19, every signal of (*S*_*p*-*D*_)-**AP5** and (*R*_*p*-*D*_)-**AP5** is different from each other, but corresponding well to the protons of **AP5**.

**Figure 2:**
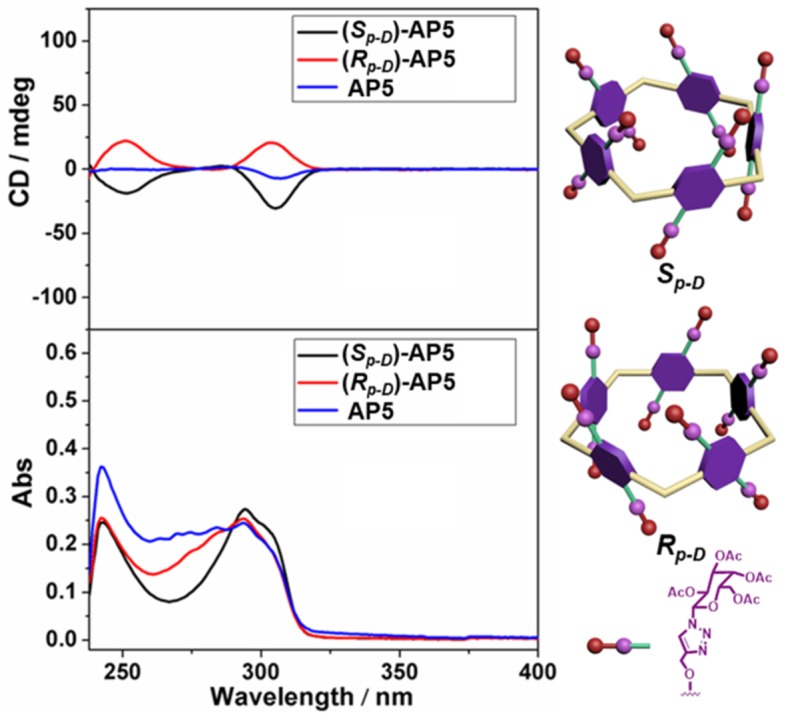
CD and UV-Vis spectra of (*S*_*p*-*D*_)-**AP5** (8 μM in CHCl_3_), (*R*_*p*-*D*_)-**AP5** (8 μM in CHCl_3_), and **AP5** (8 μM in CHCl_3_).

The circular dichroism (CD) and UV-Vis spectra of (*S*_*p*-*D*_)-**AP5**, (*R*_*p*-*D*_)-**AP5**, and **AP5** were further investigated to explain the planar chirality of **AP5**. As expected, two different kinds of CD signals could be observed between (*S*_*p*-*D*_)-**AP5** and (*R*_*p*-*D*_)-**AP5**, and **AP5** showed no obvious signal, which suggested (*S*_*p*-*D*_)-**AP5** and (*R*_*p*-*D*_)-**AP5** were mirror images in the planar chirality and they were separated effectively by silica gel chromatography (Figure 2).

**Figure 3:**
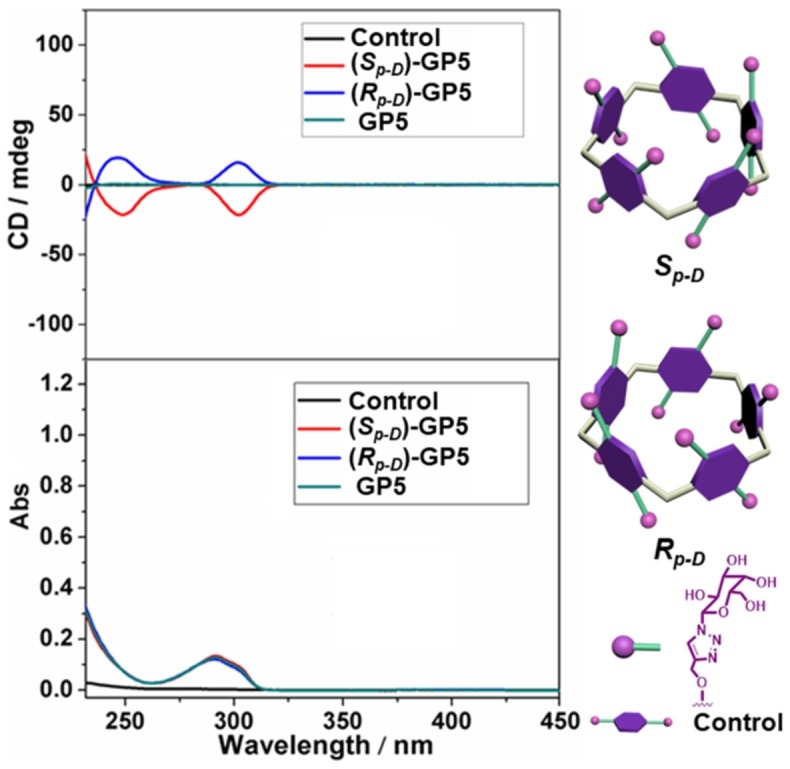
CD and UV-Vis spectra of (*S*_*p*-*D*_)-**GP5** (8 μM in H_2_O), (*R*_*p*-*D*_)-**GP5** (8 μM in H_2_O), and **GP5** (8 μM in H_2_O), and control molecule (40 μM in H_2_O).

With compounds (*S*_*p*-*D*_)-**AP5** and (*R*_*p*-*D*_)-**AP5** in hand, (*S*_*p*-*D*_)-**GP5** and (*R*_*p*-*D*_)-**GP5** were successfully obtained by reacting with sodium methoxide solution, respectively. Similar to (*S*_*p*-*D*_)-**AP5** and (*R*_*p*-*D*_)-**AP5**, ^1^H NMR and ^13^C NMR spetra of (*S*_*p*-*D*_)-**GP5** and (*R*_*p*-*D*_)-**GP5** are different. However, there is no obvious difference between (*S*_*p*-*D*_)-**GP5** and (*R*_*p*-*D*_)-**GP5** in ^1^H-^1^H COSY, NOESY, and HSQC spectra. To further investigate the planar chirality of (*S*_*p*-*D*_)-**GP5** and (*R*_*p*-*D*_)- **GP5**, CD and UV-Vis spectra were performed and two kinds of chiral signals were observed. As shown in Figure 3, the CD signals of (*S*_*p*-*D*_)-**GP5** and (*R*_*p*-*D*_)-**GP5** were fully symmetrical, which indicated (*S*_*p*-*D*_)-**GP5** and (*R*_*p*-*D*_)-**GP5** were mirror images in the planar chirality. However, no obvious CD signal could be found from **GP5**, which further confirmed (*S*_*p*-*D*_)-**GP5** and (*R*_*p*-*D*_)-**GP5** owned opposite planar chirality. For comparation, a control molecule (compound **4**) was synthesized (Scheme S2 and **Figure 10**) and no CD signal could be observed, which showed the planar chirality of pillar[5]arene was mainly attributed to the cyclization of moiety to form the pillararene backbone.

**Figure 4:**
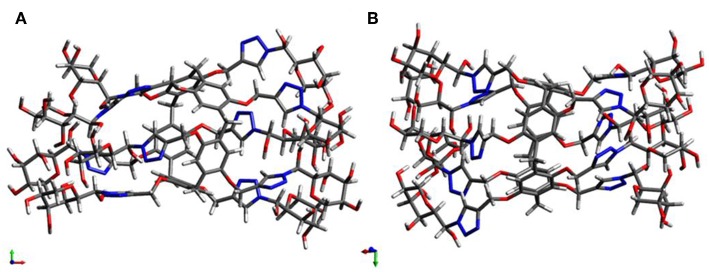
Optimized structure: **(A)** (*S*_*p*-*D*_)-**GP5** and **(B)** (*R*_*p*-*D*_)-**GP5**.

As we all know, CD spectroscopy is a well-established tool for detecting and tracking the dynamic behavior of molecule and supramolecular chirality. Pillar[5]arene derivatives could show strong CD extrema (CD_ex_) at ca. 310 nm in the absence of any other attached chromophoric groups. According to previous reports (Ogoshi et al., 2012; Yao et al., 2017), the results showed (*S*_*p*_)-pillar[5]arene derivatives exhibited negative CD_ex_ and (*R*_*p*_)-pillar[5]arene derivatives exhibited positive CD_ex_. Therefore, combining the CD spectra calculated by DFT method (Figure 4 and Figure S21), we deduced the compound with higher retention factor (R_f_) value obtained from silica gel chromatography should be the *S*_*p*_ comformer and show negative CD_ex_ signal. The compound with lower R_f_ value was the *R*_*p*_ comformer and positive CD_ex_ signal.

### Racemization Investigation of (*S*_*p*-*D*_)-GP5 and (*R*_*p*-*D*_)-GP5

**Figure 5:**
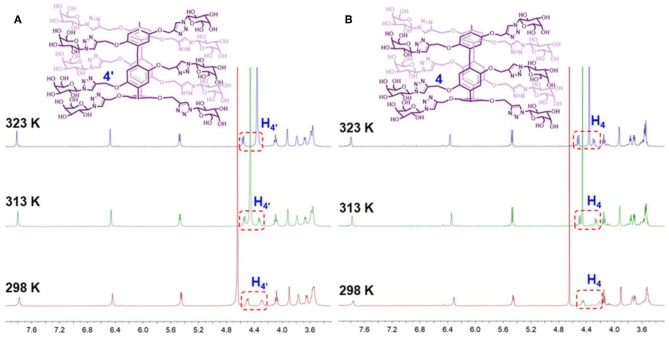
Dynamic ^1^H NMR spectra of **(A)** (*S*_*p*-*D*_)-**GP5** (4 mM in D_2_O) and **(B)** (*R*_*p*-*D*_)-**GP5** (4 mM in D_2_O).

According to previous literatures (Ogoshi et al., 2010a,b, 2011a; Nierengarten et al., 2013), the planar chirality of pillar[5]arene is unstable and will be racemized. In order to explore whether (*S*_*p*-*D*_)-**GP5** and (*R*_*p*-*D*_)-**GP5** could exchange with each other, dynamic ^1^H NMR and CD measurements were further carried out. According to the planar chirality of (*S*_*p*-*D*_)-**GP5** and (*R*_*p*-*D*_)-**GP5**, the two protons from the methylene moieties adjacent to the O atoms (H_4_) were different in chemical environment and could split into two groups of double peak in 1:1 integration ratio at 298 K (Figure S20). Thus, the split proton resonances are a useful marker to determine whether the rotation of pillar[5]arenes takes place on the NMR time scale (Ogoshi et al., 2010a,b, 2011b). Moreover, as shown in Figure 5, although the chemical shift of D_2_O exhibited upfield shift changes due to the weakening of intermolecular hydrogen bonding of D_2_O with increasing temperature, almost no peak changes for (*S*_*p*-*D*_)-**GP5** and (*R*_*p*-*D*_)-**GP5** could be observed (TMS as the reference). More important, the split of ${\text{H}}_{4^{\text{′}}}$ and H_4_ still retained during the progress of heating, indicating (*S*_*p*-*D*_)-**GP5** and (*R*_*p*-*D*_)-**GP5** were stable and hardly racemized on the NMR time scale in the measured temperature range.

**Figure 6:**
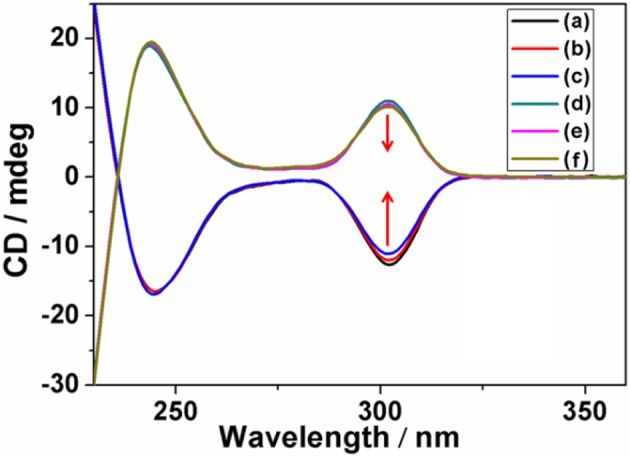
Dynamic CD spectra of (*S*_*p*-*D*_)-**GP5** [**(A–C)** 8 μM in H_2_O] and (*R*_*p*-*D*_)-**GP5** [**(D–F)** 8 μM in H_2_O] at 298, 313, and 323 K, respectively.

Subsequently, dynamic CD experiments were investigated, and the results indicated the intensity of (*S*_*p*-*D*_)-**GP5** and (*R*_*p*-*D*_)-**GP5** were stable and symmetric, confirming the planar chirality of (*S*_*p*-*D*_)-**GP5** and (*R*_*p*-*D*_)-**GP5** was absolutely independent and the racemization of (*S*_*p*-*D*_)-**GP5** and (*R*_*p*-*D*_)-**GP5** didn't happen even under higher temperature. Whereas, when more attention was paid to the wavelength from 290 to 310 nm, which was ascribed to π-π^*^ transitions of the aromatic moieties in the pillar[5]arene backbone, both (*S*_*p*-*D*_)-**GP5** and (*R*_*p*-*D*_)-**GP5** trended to racemize with increasing temperature (Figure 6 and Figure S22). However, due to the large molecular size of bulky substituent on the rim of **GP5**, neither (*S*_*p*-*D*_)-**GP5** nor (*R*_*p*-*D*_)-**GP5** could racemize actually, which is consistent with the dynamic ^1^H NMR results (Ogoshi et al., 2011a, 2016). In summary, different from many traditional pillar[5]arene derivatives, the planar chirality of (*S*_*p*-*D*_)-**GP5** and (*R*_*p*-*D*_)-**GP5** are very stable and unchangeable, which can be used as a reliable chiral source to induce and transfer the *S*_*p*_ and *R*_*p*_ planar chirality.

Simultaneously, after dialysis with distilled water, the CD spectra of the (*S*_*p*-*D*_)-nanoparticles and (*R*_*p*-*D*_)-nanoparticles were obtained, respectively. The results confirmed the planar chirality of these chiral nanoparticles still existed and displayed symmetrical signal, indicating that (*S*_*p*-*D*_)-**GP5** and (*R*_*p*-*D*_)-**GP5** could be used as reliable chiral sources to transfer the *S*_*p*_ and *R*_*p*_ planar chirality (**Figure 8**).

### The Construction of Chiral Nanoparticles

**Figure 7:**
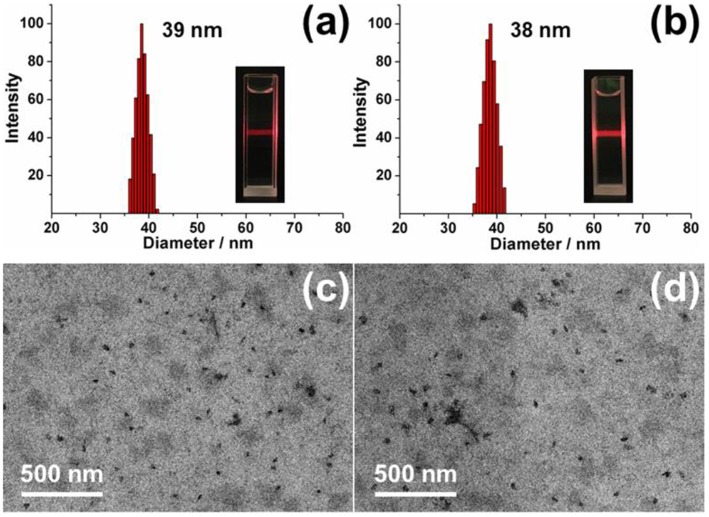
DLS data of chiral nanoparticles: **(a)** (*S*_*p*-*D*_)-nanoparticles. Inset photo: Tyndall effect. **(b)** (*R*_*p*-*D*_)-nanoparticles. Inset photo: Tyndall effect. TEM images of chiral nanoparticles: **(c)** (*S*_*p*-*D*_)-nanoparticles. **(d)** (*R*_*p*-*D*_)-nanoparticles.

Based on the outstanding host-guest properities of pillar[5]arene, one of our previously reported guest molecule (**DNS-CPT**) (Sun et al., 2019) was used to investigate the construction of nanoparticles with planar chirality (**Figure 11**). As shown in Figure 7, when (*S*_*p*-*D*_)-**GP5** or (*R*_*p*-*D*_)-**GP5** was added into the **DNS-CPT** solution, an obvious Tyndall effect could be observed, indicating the formation of large sized aggregates. The diameter of these nanoparticles was confirmed to be 39 and 38 nm by dynamic light scattering (DLS), respectively. The morphology of the nanoparticles was further investigated by transmission electron microscopy (TEM), and the results showed both (*S*_*p*-*D*_)- and (*R*_*p*-*D*_)-**GP5** could form nanoparticles with the presence of the guest molecule **DNS-CPT** (Figure 7 and Figure S23). Moreover, Zeta potential measurements showed that the obtained nanoparticles possess relatively high positive ζ- potentials (32.85 and 35.93 mV, respectively), suggesting their good stability in solution (Figure S24).

**Figure 8:**
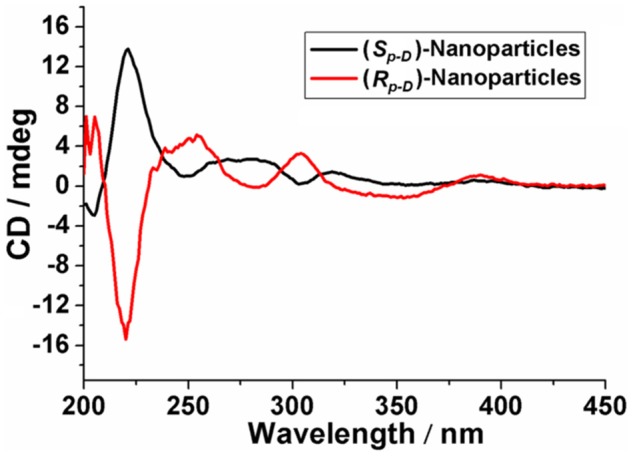
CD spectrum of chiral nanoparticles.

## Experimental

### Synthesis of GP5

**Figure 9:**
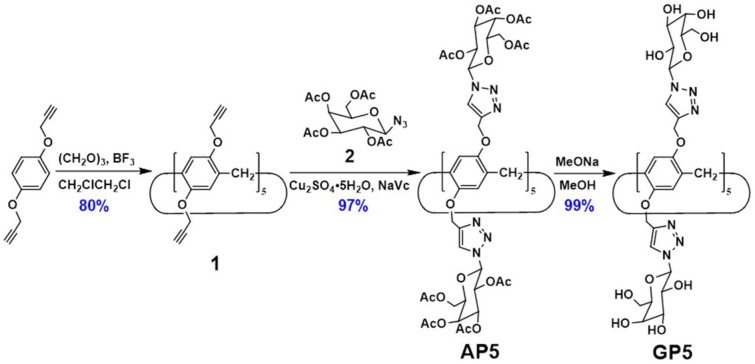
Synthesis route of **GP5**.

As shown in Figure 9, **GP5** was synthesized based on the click reaction between compound **1** and **2** to generate compound **AP5** successfully. Then, **AP5** was reacted with sodium methoxide in methanol for 24 h under an inert atmosphere at ambient temperature. The resulting reaction mixture was filtered and washed with methanol, which gave the target macrocycle **GP5** in 99% yield. A combination of ^1^H, ^13^C, ^1^H-^1^H COSY, NOESY, and HSQC nuclear magnetic resonance spectroscopy (NMR) confirmed (Figures S7–S16) the formation of **GP5**.

**Figure 10:**
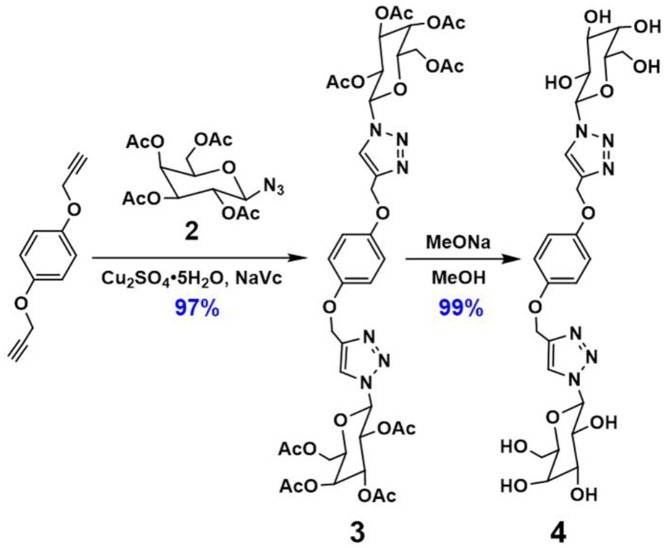
Synthesis route of compound **4**.

**Figure 11:**
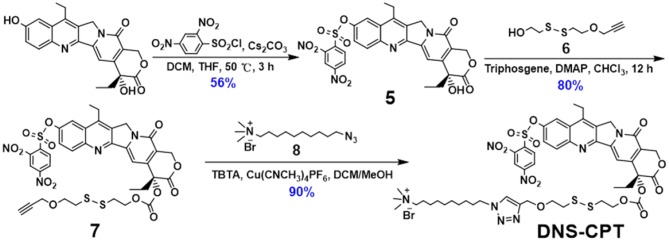
Synthesis route of **DNS-CPT**.

### Synthesis of Compound 4

Compound **4** was synthesized based on the click reaction between 1,4-bis(prop-2-yn-1-yloxy)benzene and compound **2** to generate compound **3** successfully. Then, compound **3** was reacted with sodium methoxide in methanol for 24 h under an inert atmosphere at ambient temperature. The resulting reaction mixture was filtered and washed with methanol, which gave the control molecule compound **4** in 99% yield. ^1^H NMR (Figures S17, S18) confirmed the formation of compounds **3** and **4**.

### Synthesis of DNS-CPT

**DNS-CPT** was synthesized and characterized according to our previous work (Sun et al., 2019).

### Fourier Transform Infrared Spectrometer (FT-IR) Spectrum

**Figure 12:**
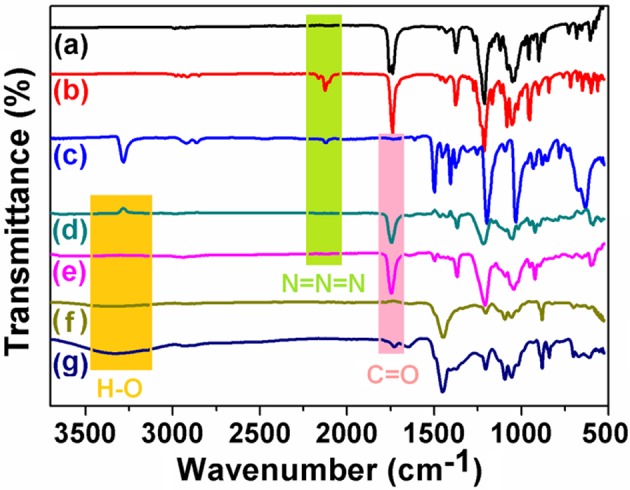
FT-IR spectrum: **(a)** Penta-O-acetyl-β-D-galactopyranose, **(b)** Compound **2**, **(c)** Compound **1**, (*S*_*p*-*D*_)-**AP5**, **(e)** (*R*_*p*-*D*_)-**AP5**, **(f)** (*S*_*p*-*D*_)-**GP5**, and **(g)** (*R*_*p*-*D*_)-**GP5**, respectively.

FT-IR experiments of 1,2,3,4,6-penta-o-acetyl-β-D-galactopyranose, compound **1**, compound **2**, (*S*_*p*-*D*_)-**AP5**, (*R*_*p*-*D*_)-**AP5**, (*S*_*p*-*D*_)-**GP5**, and (*R*_*p*-*D*_)-**GP5** were carried out to track the functionalization process of pillar[5]arene. As shown in Figure 12, after reaction with trimethylsilyl azide (TMS-N_3_), a typical N = N = N peak at 2,100 cm^−1^ could be observed. However, the N = N = N peak disappeared after the click reaction with compound **1**, which indicated the 1,2,3,4,6-penta-o-acetyl-β-D-galactopyranose group had been modified to pillar[5]arene to obtain **AP5** successfully. Meanwhile, the stretching vibration peak of C = O was detected at 1750 cm^−1^. Moreover, when the acetyl group of **AP5** was removed, the characteristic absorption peak of C = O disappeared and a wide peak of O-H at 3300 cm^−1^ was observed at the same time, which showed the successful formation of **GP5**.

## Conclusion

In conclusion, we successfully obtained (*S*_*p*-*D*_)-**AP5**, (*R*_*p*-*D*_)-**AP5**, (*S*_*p*-*D*_)-**GP5**, and (*R*_*p*-*D*_)-**GP5** through silica gel chromatography with a high yield at room temperature. Dynamic CD and ^1^H NMR experiments revealed the *S*_*p*_ and *R*_*p*_ planar chirality of these pillar[5]arene derivatives (**GP5**) were very stable and unracemized, which could be used as reliable chiral sources to construct chiral nanoparticles, showing the *S*_*p*_ and *R*_*p*_ planar chirality of **GP5** could be transferred by the host-guest interaction based on **GP5** and **DNS-CPT**.

## Data Availability Statement

All datasets generated for this study are included in the article/Supplementary Material.

## Author Contributions

LW, X-YH, and JJ conceived the project, supervised the study, and revised the manuscript. GS conducted the experiments, wrote the draft manuscript, and prepared the supporting information. LP conducted the TEM experiments. SP conducted the calculation experiments. All authors analyzed and interpreted the data.

### Conflict of Interest

The authors declare that the research was conducted in the absence of any commercial or financial relationships that could be construed as a potential conflict of interest.
