# Supplementary material for: β-D-Galactose-Functionalized Pillar[5]arene With Interesting Planar-Chirality for Constructing Chiral Nanoparticles
Source: Front Chem. 2019 Nov 14;7:743. doi: 10.3389/fchem.2019.00743 (PMC6869513; doi:10.3389/fchem.2019.00743)
Supplement: Supplementary file 1 [file Data_Sheet_1.pdf]

Supporting Information

β-D-Galactose-Functionalized Pillar[5]arene with Interesting Planar-Chirality for Constructing Chiral Nanoparticles

Guangping Sun[1], Liangtao Pu[4], Srikala Pangannaya[1], Tangxin Xiao[3], Xiao-Yu Hu[1,2*], Juli Jiang[1*], and Leyong Wang[1,3]

[1]Key Laboratory of Mesoscopic Chemistry of MOE, Jiangsu Key Laboratory of Advanced Organic Materials, School of Chemistry and Chemical Engineering, Nanjing University, Nanjing 210023, China

[2]Applied Chemistry Department, College of Material Science and Technology, Nanjing University of Aeronautics and Astronautics, Nanjing 211100, China

[3]School of Petrochemical Engineering, Changzhou University, Changzhou 213164, China.

[4]State Key Laboratory of Pollution Control and Resource Reuse, School of Environment, Nanjing University, Nanjing 210023, China.

***Correspondence:**
Xiao-Yu Hu; Juli Jiang
huxy@nuaa.edu.cn (X.-Y. Hu); jjl@nju.edu.cn (J.Jiang)

Table of Contents

1. General information S2

2. Synthesis of GP5 .... S2

3. Synthesis of control molecule S12

4. ^{1}H NMR spectra of (S_{p-D})-AP5, (R_{p-D})-AP5, and AP5 .... S14

5. ^{1}H NMR spectra of (S_{p-D})-GP5 and (R_{p-D})-GP5 .... S14

6. Calculated CD spectra of (S_{p-D})-GP5 and (R_{p-D})-GP5 .... S15

7. Dynamic CD spectra of (S_{p-D})-GP5 and (R_{p-D})-GP5 .... S15

8. Enlarge TEM images of nanoparticles S15

9. Zeta potential of nanoparticles S16

10. References S16

1. General information

All reactions were performed in air atmosphere unless otherwise stated. The commercially available reagents and solvents were either employed as purchased or dried according to procedures described in the literature. Column chromatography was performed with silica gel (200-300 and 300-400 mesh) produced by Qingdao Marine Chemical Factory, Qingdao (China). All yields were given as isolated yields. NMR spectra were recorded on a Bruker DPX 400 MHz spectrometer (or Bruker DPX 500 MHz spectrometer) with internal standard tetramethyl silane (TMS) and solvent signals as internal references at room temperature, and the chemical shifts (δ) were expressed in ppm and J values were given in Hz. High-resolution electrospray ionization mass spectra (HR-ESI-MS) were recorded on an Agilent 6540Q-TOF LCMS equipped with an electrospray ionization (ESI) probe operating in the positive-ion mode with direct infusion. Low-resolution electrospray ionization mass spectra (LR-ESI-MS) were obtained on Finnigan Mat TSQ 7000 instruments. The UV-Vis absorption spectra were measured on a Perkin Elmer Lambda 35 UV-Vis Spectrometer. CD spectra were measured by J-810 Circular Dichroism Spectrometer, JASCO (China). Transmission electron microscope (TEM) investigations were carried out on a JEM-2100 instrument. Dynamic light scattering (DLS) measurements were carried out on a Brookhaven BI-9000AT system (Brookhaven Instruments Corporation, USA), using a 200-mW polarized laser source (λ = 514 nm). Zeta-potential measurements were performed at 25 ℃ on a Zetasizer Nano Z apparatus (Malvern Instruments Ltd., UK) using the Smoluchowski model for the calculation of the Zeta-potential from the measured electrophoretic mobility. FT-IR spectra were measured on Nicolet iS5 (Thermo Fisher Scientific, USA).

2. Synthesis of GP5

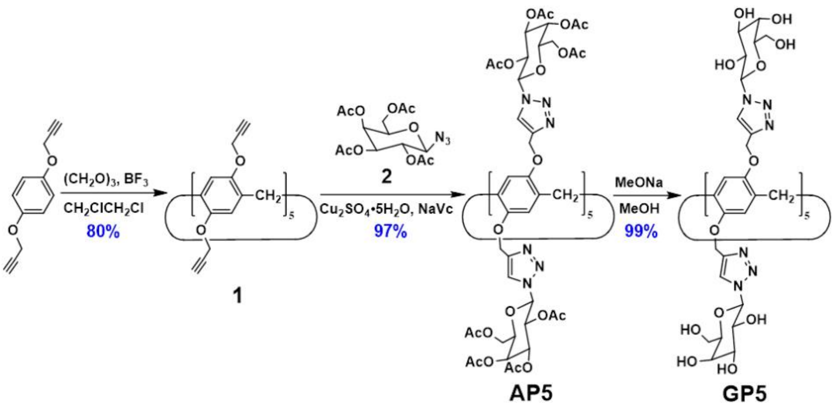

Scheme S1. Synthesis route of **GP5**.

Synthesis of compound 1

Compound **1** was prepared according to previously reported method.[S1]

1,4-Bis(prop-2-yn-1-yloxy)benzene (3.72 g, 20 mmol) and paraformaldehyde (1.40 g, 40 mmol) were dissolved in 1,2-dichloroethane (60 mL). Then boron trifluoride diethyl etherate (4 mL) was added to the solution and the mixture was stirred at room temperature for 24 h. Methanol (240 mL) was added to quench the reaction. The mixture was filtered and the crude product was purified by silica gel chromatography using dichloromethane as fluent to give compound **1** as a white solid

(3.17 g, 3.2 mmol, 80 %). ^{1}H NMR (400 MHz, $CDCl_3$, 298 K) δ (ppm) = 6.82 (s, 10H), 4.53 (d, *J* = 2.4 Hz, 20H), 3.81 (s, 10H), 2.31 (t, *J* = 2.4 Hz, 10H).

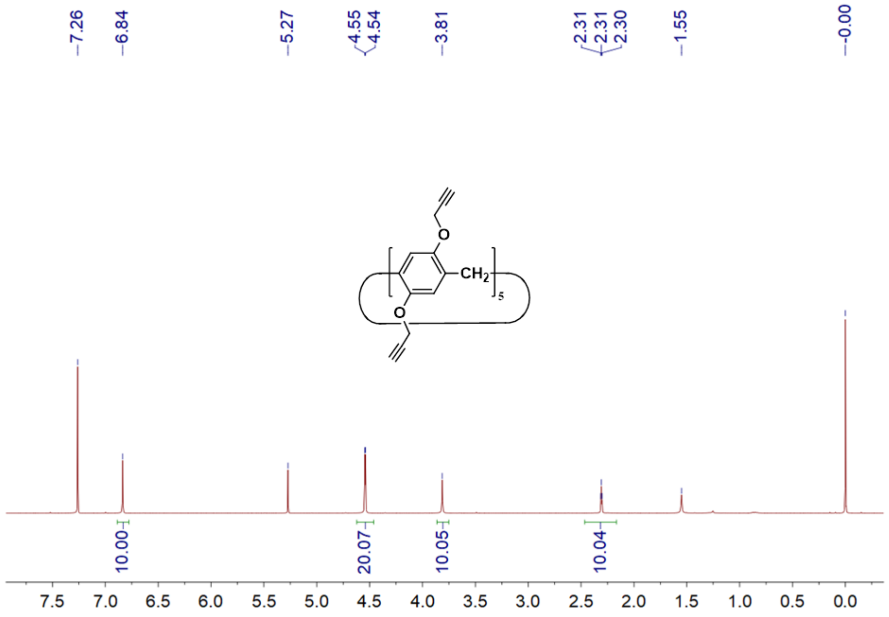

Figure S1. ^{1}H NMR spectrum (400 MHz, $CDCl_3$, 298 K) of compound **1**.

Synthesis of compound 2

Compound **2** was prepared according to previously reported method.[S2]

1,2,3,4,6-Penta-O-acetyl-β-D-galactopyranose (1.36 g, 34.9 mmol) was dissolved in anhydrous CH_2Cl_2 (14 mL) under argon. Trimethylsilyl azide (1.61 g, 1.85 mL, 13.9 mmol) and tin(IV) chloride (0.24 g, 0.11 mL, 0.91 mmol) was added. After stirring for 12 h at room temperature TLC (cyclohexane/toluene/ethylacetate=3:3:1, *v/v/v*) showed complete consumption of the starting material. The reaction mixture was washed successively with saturated aqueous $NaHCO_3$ solution (10 mL), water (10 mL) and brine (10 mL). The organic layer was dried over anhydrous Na_2SO_4. The solvent was removed and the residue was purified by silica gel chromatography using (cyclohexane/ethyl acetate= 3:1, *v/v*) as fluent to afford compound **2** (1.29 g, 3.45 mmol, 99%) as a white solid. ^{1}H NMR (400 MHz, $CDCl_3$, 298 K) *δ* (ppm) = 5.43 (dd, *J* = 3.2, 0.8 Hz, 1H), 5.17 (dd, *J* = 10.4, 8.8 Hz, 1H), 5.05 (dd, *J* = 10.4, 3.6 Hz, 1H) , 4.61 (d, *J* =8.8 Hz, 1H) , 4.21-4.13 (m, 2H) , 4.01 (td, *J* = 7.2, 0.8 Hz, 1H), 2.17 (s, 3H), 2.09 (s, 3H), 2.07 (s, 3H), 1.99 (s, 3H).

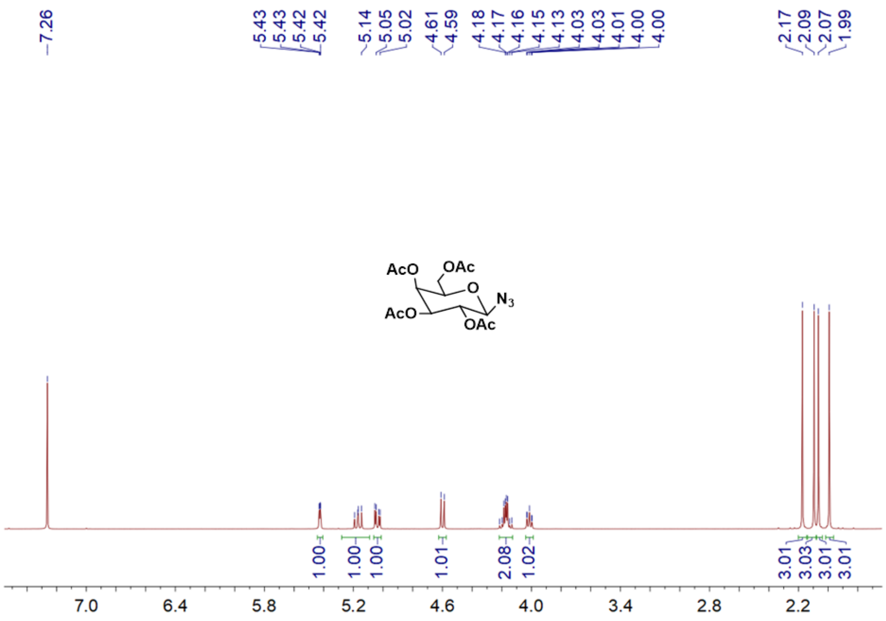

Figure S2. ^{1}H NMR spectrum (400 MHz, $CDCl_3$, 298 K) of compound **2**.

Synthesis of compound AP5

Compound **2** (0.5 g, 1.34 mmol), copper sulfate pentahydrate (0.03 g, 0.12 mmol) and sodium ascorbate (0.08 g, 0.57 mmol) were added to a solution of compound **1** (0.07 g, 0.07 mmol) in dichloromethane (10 mL). Then 10 mL H_2O was added. The mixture was stirred under argon atmosphere in room temperature for 24 h. The reaction mixture was diluted with dichloromethane (10 mL) and washed with water (30 mL). The organic phase was dried over magnesium sulfate and filtered. The solvent was removed and the crud product was purified by silica gel chromatography (dichloromethane/methanol = 40:1, *v/v*) afforded ($S_{p\text{-}D}$)-**AP5** (0.18 g, 0.04 mmol, 54%) and ($R_{p\text{-}D}$)-**AP5** (0.14 g, 0.03 mmol, 43%) as white solid. ($S_{p\text{-}D}$)-**AP5**: ^{1}H NMR (400 MHz, $CDCl_3$, 298 K) δ (ppm) = 8.19 (s, 10H), 6.76 (s, 10H), 5.94 (d, J = 9.2 Hz, 10H), 5.64 (t, J = 9.6 Hz, 10H), 5.55(d, J = 3.2 Hz, 10H), 5.37 (dd, J = 10.4, 3.2 Hz, 10H), 4.87 (d, J = 11.6 Hz, 10H), 4.83 (d, J = 11.6 Hz, 10H), 4.39-4.15 (m, 30H), 3.75 (s, 10H), 2.23 (s, 30H), 2.01 (s, 30H), 1.99 (s, 30H), 1.86 (s, 30H). ^{13}C NMR (100 MHz, $CDCl_3$, 298 K) δ (ppm) = 170.5, 170.2, 170.0, 169.1, 149.3, 145.0, 127.8, 122.2, 114.3, 85.8, 73.4, 70.8, 68.4, 66.9, 61.7, 60.8, 29.3, 20.8, 20.6, 20.5, 20.3. ($R_{p\text{-}D}$)-**AP5**: ^{1}H NMR (400 MHz, $CDCl_3$, 298 K) δ (ppm) = 8.09 (s, 10H), 6.93 (s, 10H), 6.02 (d, J = 9.2 Hz, 10H), 5.74 (t, J = 9.6 Hz, 10H), 5.54(d, J = 3.2 Hz, 10H), 5.27 (dd, J = 10.4, 3.2 Hz, 10H), 5.07 (d, J = 11.6 Hz, 10H), 4.88 (d, J = 11.6 Hz, 10H), 4.29-4.10 (m, 30H), 3.79 (s, 10H), 2.22 (s, 30H), 2.03 (s, 30H), 2.00 (s, 30H), 1.76 (s, 30H). ^{13}C NMR (100 MHz, $CDCl_3$, 298 K) δ (ppm) = 170.3, 170.2, 169.9, 168.9, 149.8, 145.0, 128.7, 122.3, 115.3, 85.8, 73.4, 71.0, 68.4, 66.9, 62.0, 60.9, 29.4, 20.7, 20.7, 20.5, 20.2.

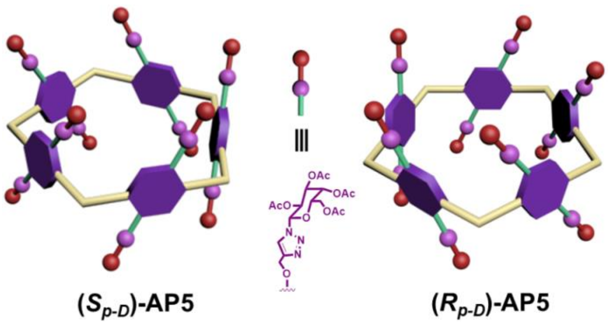

Figure S3. Structures of ($S_{p\text{-}D}$)-**AP5** and ($R_{p\text{-}D}$)-**AP5**.

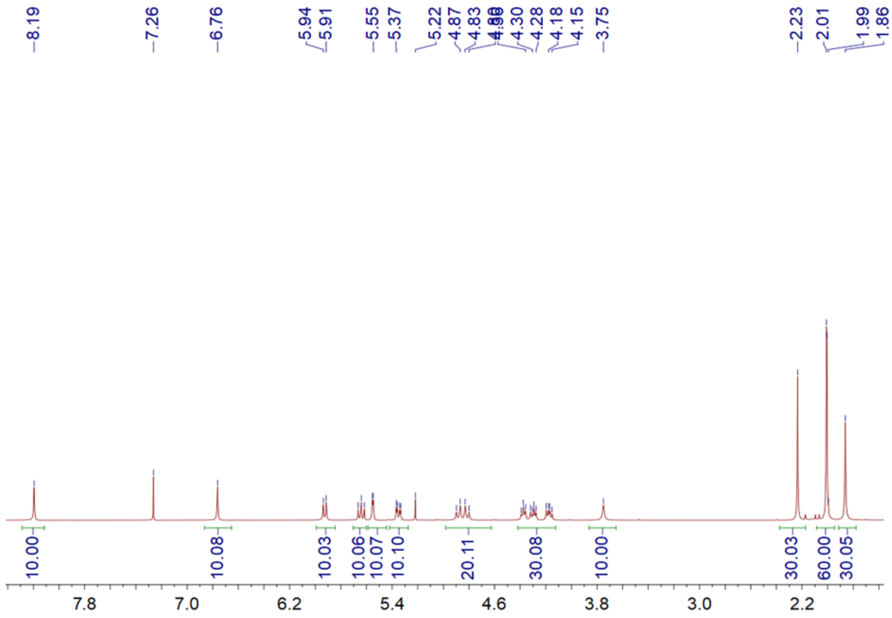

Figure S4. ^{1}H NMR spectrum (400 MHz, $CDCl_3$, 298 K) of ($S_{p\text{-}D}$)-**AP5**.

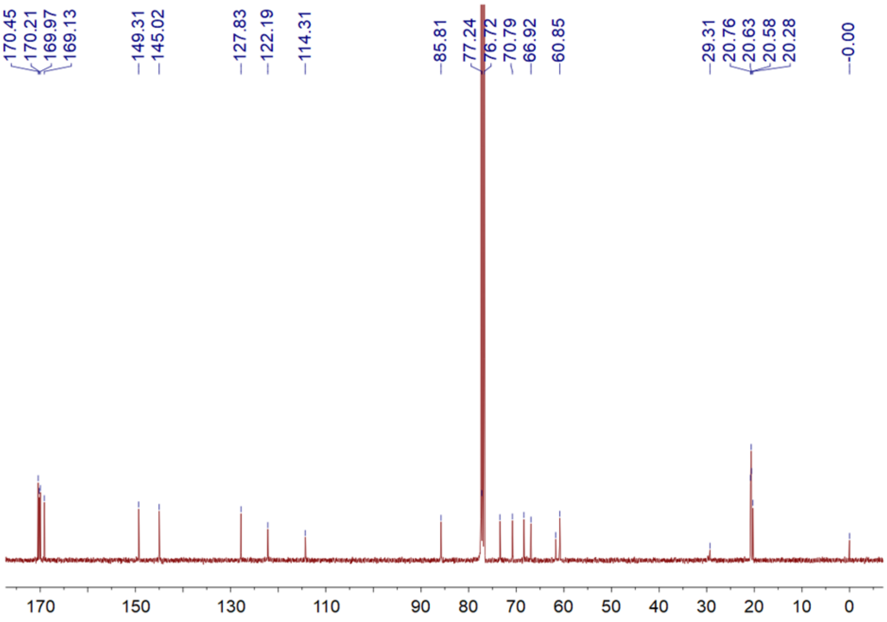

Figure S5. ^{13}C NMR spectrum (400 MHz, $CDCl_3$, 298 K) of ($S_{p\text{-}D}$)-**AP5**.

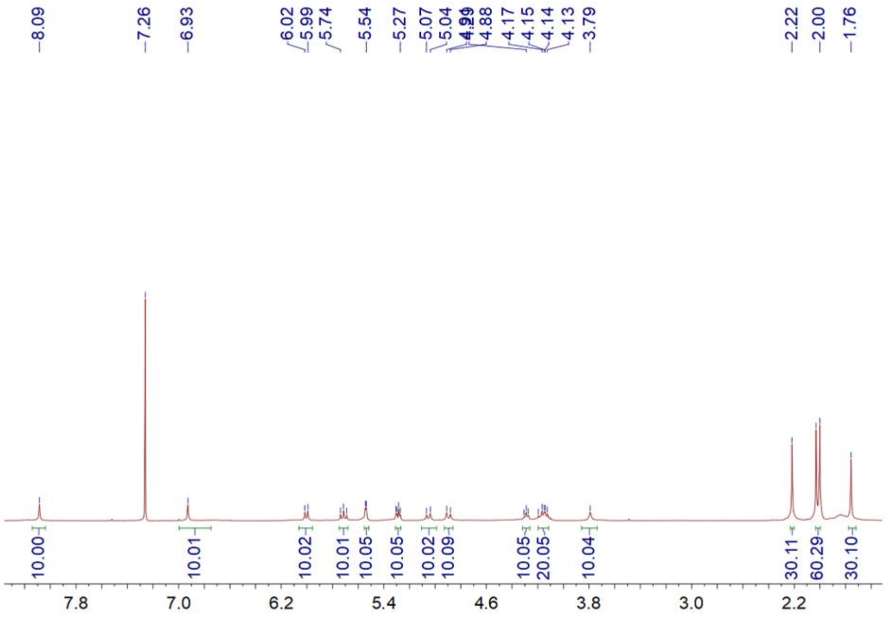

Figure S6. ^{1}H NMR spectrum (400 MHz, $CDCl_3$, 298 K) of ($R_{p\text{-}D}$)-**AP5**.

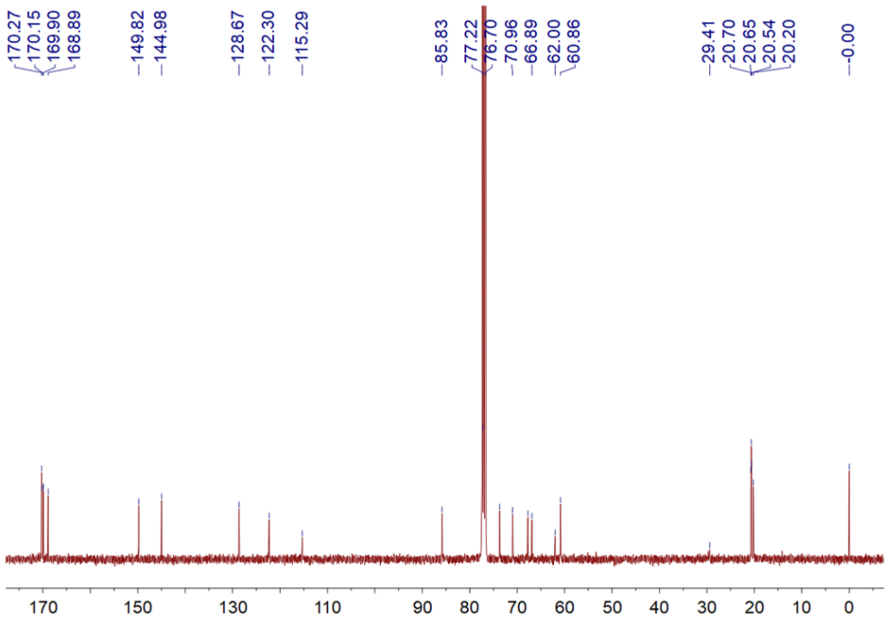

Figure S7. ^{13}C NMR spectrum (400 MHz, $CDCl_3$, 298 K) of ($R_{p\text{-}D}$)-**AP5**.

Synthesis of compound GP5

Compound **AP5** (0.9 g, 0.19 mmol) was dissolved in a sodium methoxide solution (35 mL, 0.15 M) in a clean dry round bottom flask. The solution was stirred for 12 h. The precipitate was filtered under suction and washed with CH_3OH (5 × 20 mL) to give **GP5** (0.57 g, 0.19 mmol, 99%) as a white solid. ($S_{p\text{-}D}$)-**GP5**: ^{1}H NMR (400 MHz, D_2O, 298 K) δ (ppm) = 7.92 (s, 10H), 6.58 (s, 10H), 5.60 (d, J = 8.8 Hz, 10H), 4.62 (d, J = 11.6 Hz, 10H), 4.41 (d, J = 11.6 Hz, 10H), 4.20 (t, J = 9.2 Hz, 10H), 4.04 (d, J = 2.8 Hz, 10H), 3.90 (t, J = 5.2 Hz, 10H), 3.72 (dd, J = 9.6, 2.4 Hz, 10H),

3.69-3.65 (m, 30H). ^{13}C NMR (100 MHz, D_2O, 298 K) δ (ppm) = 150.2, 143.5, 129.2, 124.5, 117.3, 88.0, 78.2, 73.0, 69.7, 68.5, 62.1, 60.6, 31.1. ($R_{p\text{-}D}$)-**GP5**: ^{1}H NMR (400 MHz, D_2O, 298 K) δ (ppm) = 7.91 (s, 10H), 6.45 (s, 10H), 5.59 (d, J = 8.8 Hz, 10H), 4.56 (d, J = 11.6 Hz, 10H), 4.35 (d, J = 11.6 Hz, 10H), 4.28 (t, J = 9.2 Hz, 10H), 4.03 (d, J = 2.8 Hz, 10H), 3.89-3.85 (m, 20H), 3.70-3.61 (m, 30H). ^{13}C NMR (100 MHz, D_2O, 298 K) δ (ppm) = 150.3, 143.5, 129.1, 124.6, 117.0, 88.0, 78.0, 73.0, 69.5, 68.4, 62.0, 60.5, 31.2.

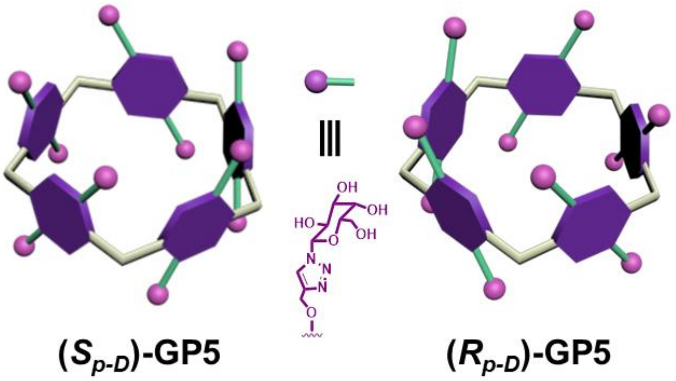

Figure S8. Structures of ($S_{p\text{-}D}$)-**GP5** and ($R_{p\text{-}D}$)-**GP5**.

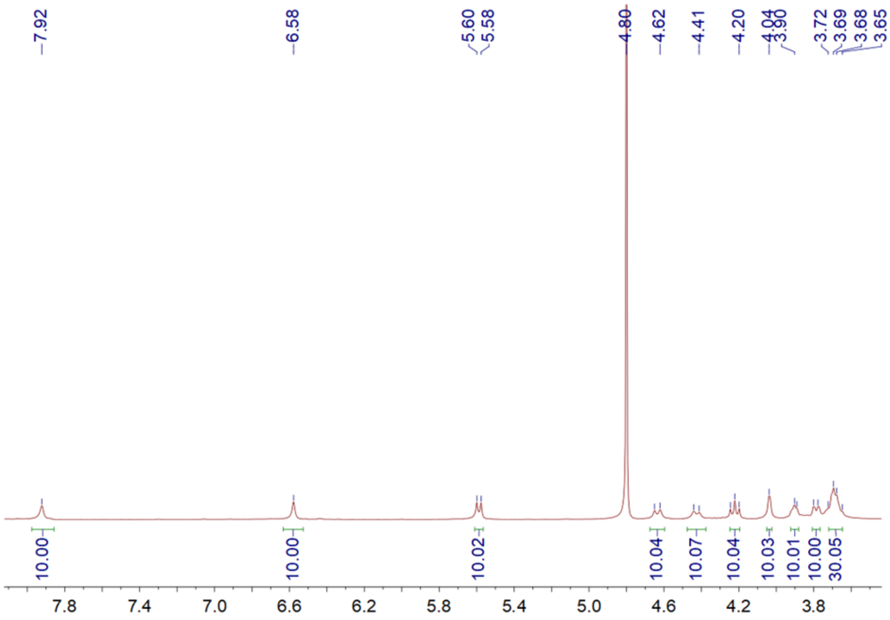

Figure S9. ^{1}H NMR spectrum (400 MHz, D_2O, 298 K) of ($S_{p\text{-}D}$)-**GP5**.

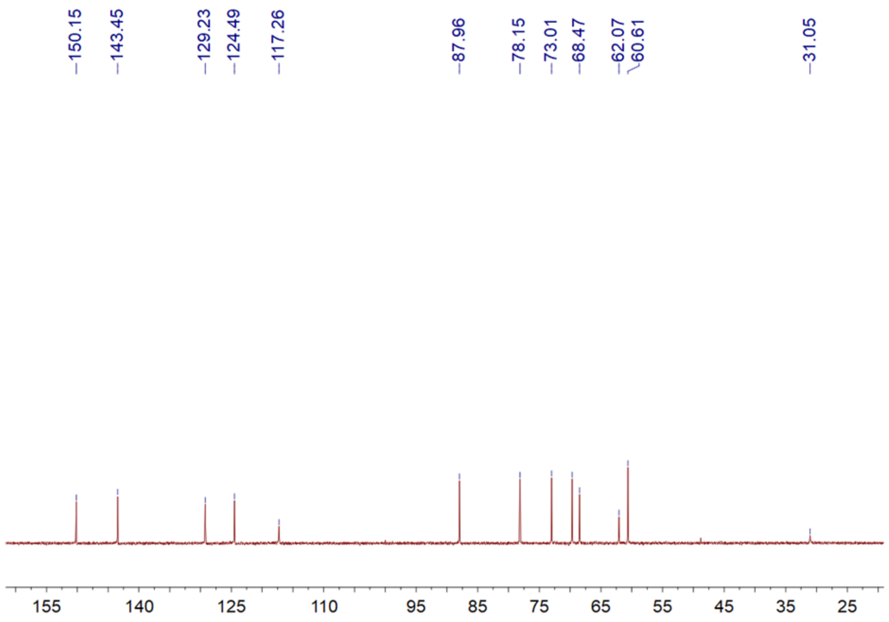

Figure S10. ^{13}C NMR spectrum (400 MHz, D_2O, 298 K) of ($S_{p\text{-}D}$)-**GP5**.

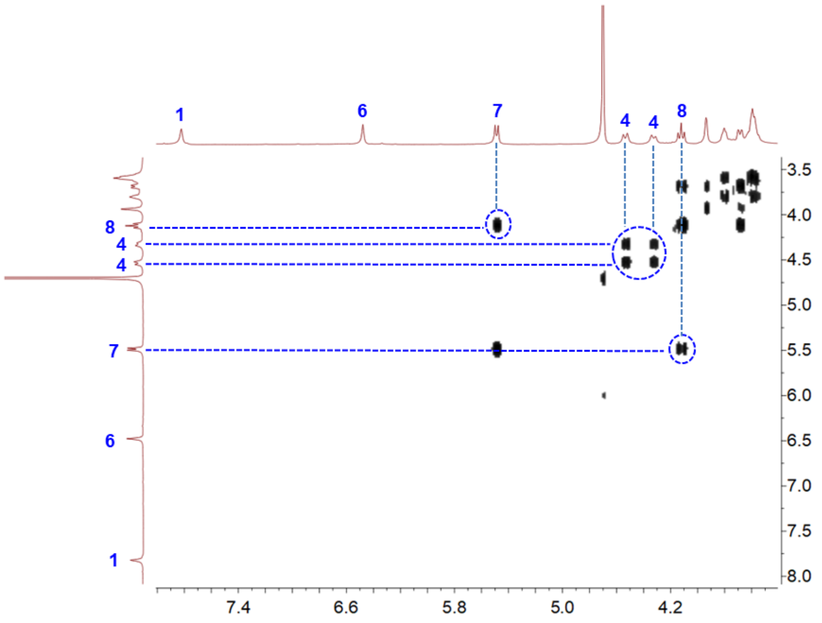

Figure S11. ^{1}H-^{1}H COSY spectrum (400 MHz, D_2O, 298 K) of ($S_{p\text{-}D}$)-**GP5**.

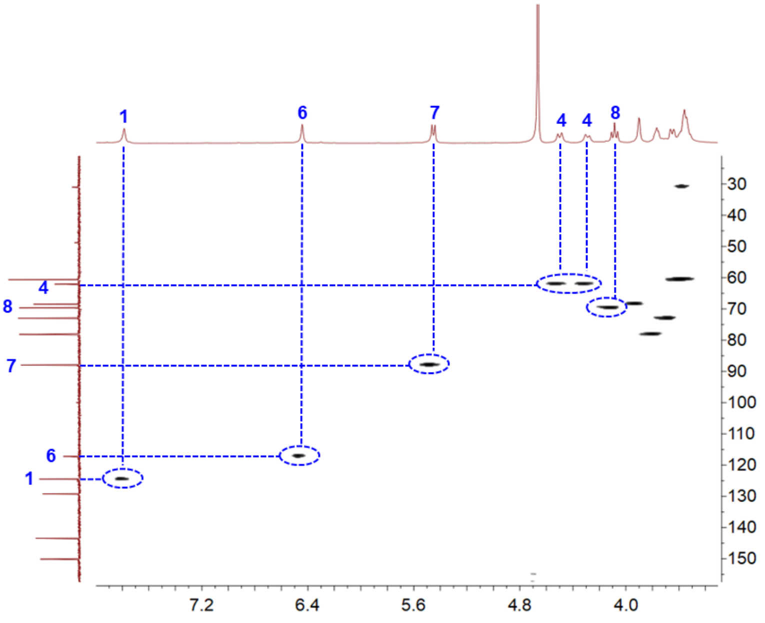

Figure S12. HSQC spectrum (400 MHz, D_2O, 298 K) of ($S_{p\text{-}D}$)-**GP5**.

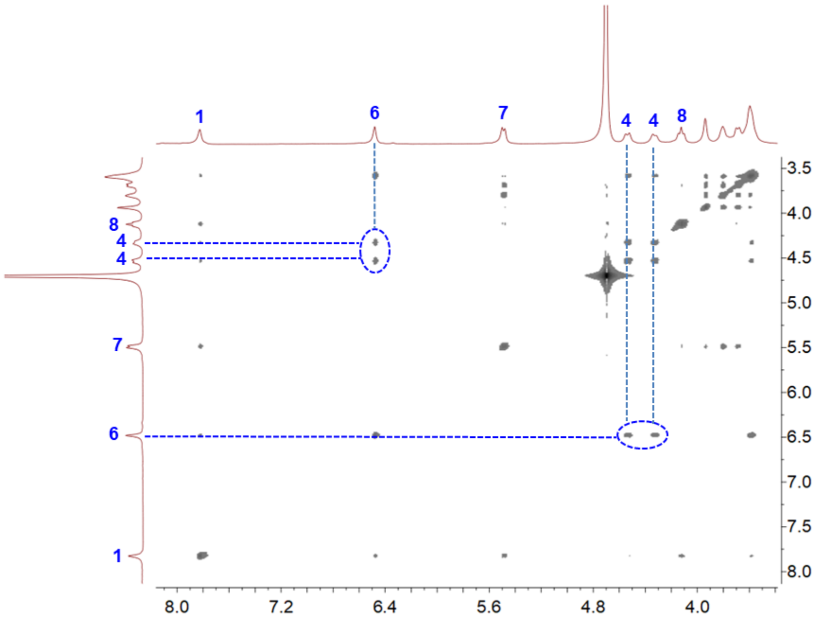

Figure S13. NOESY spectrum (400 MHz, D_2O, 298 K) of ($S_{p\text{-}D}$)-**GP5**.

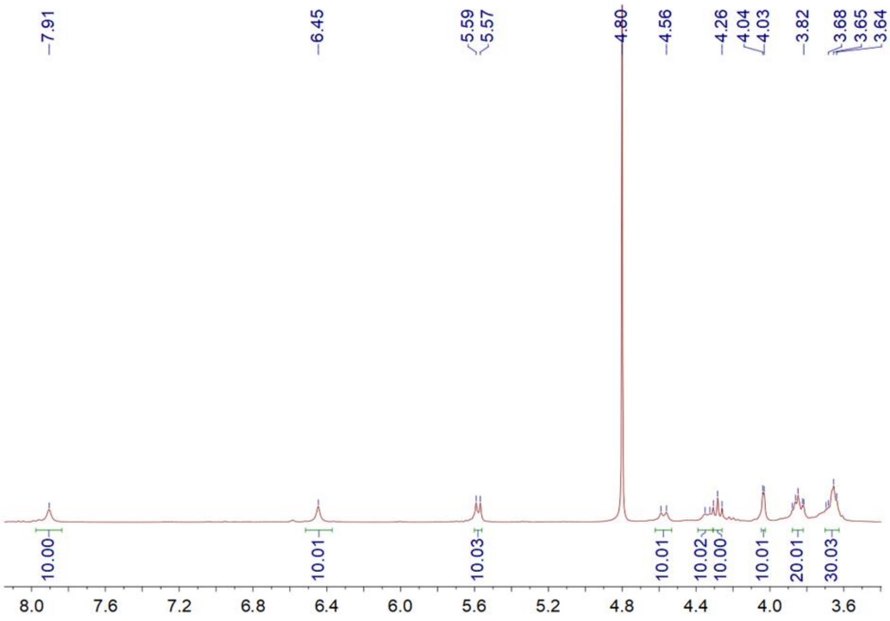

Figure S14. ^{1}H NMR spectrum (400 MHz, D_2O, 298 K) of ($R_{p\text{-}D}$)-**GP5**.

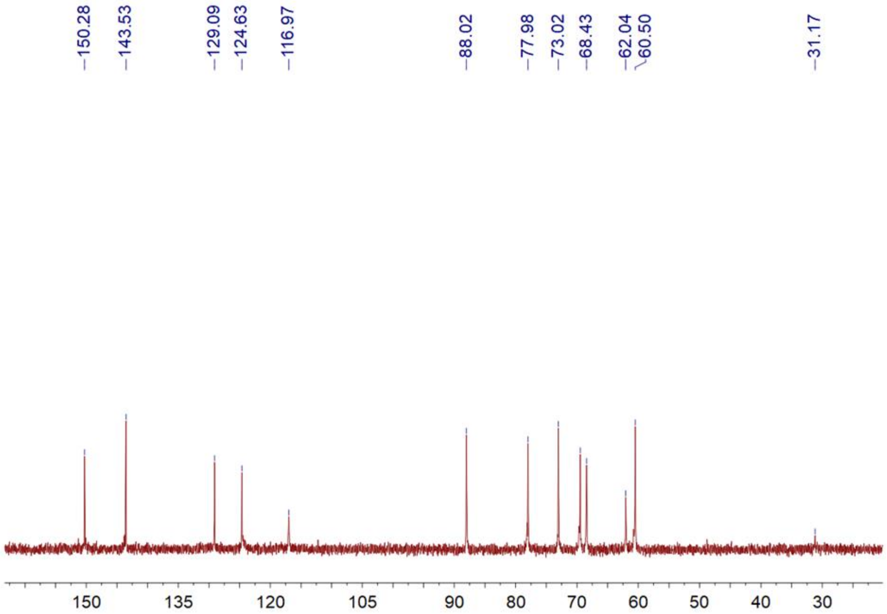

Figure S15. ^{13}C NMR spectrum (400 MHz, D_2O, 298 K) of ($R_{p\text{-}D}$)-**GP5**.

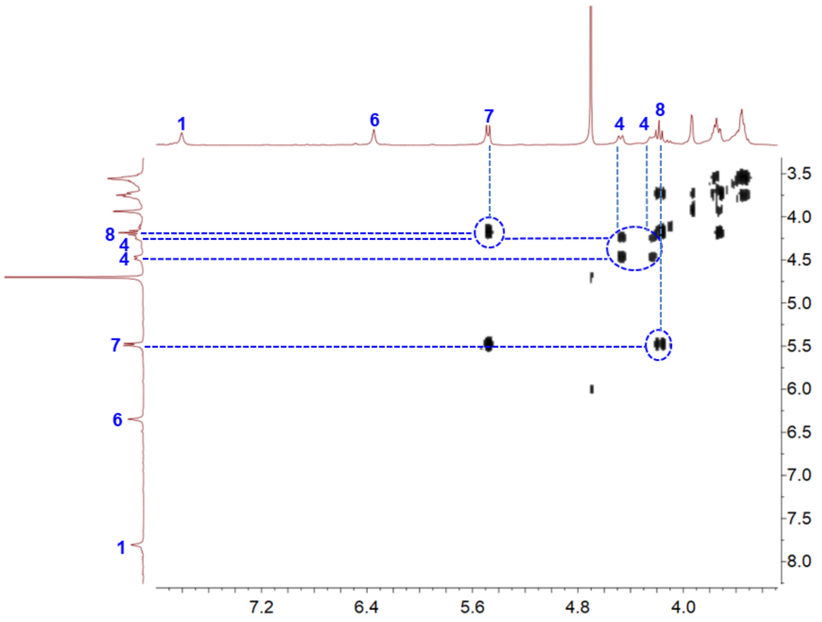

Figure S16. ^{1}H-^{1}H COSY spectrum (400 MHz, D_2O, 298 K) of ($R_{p\text{-}D}$)-**GP5**.

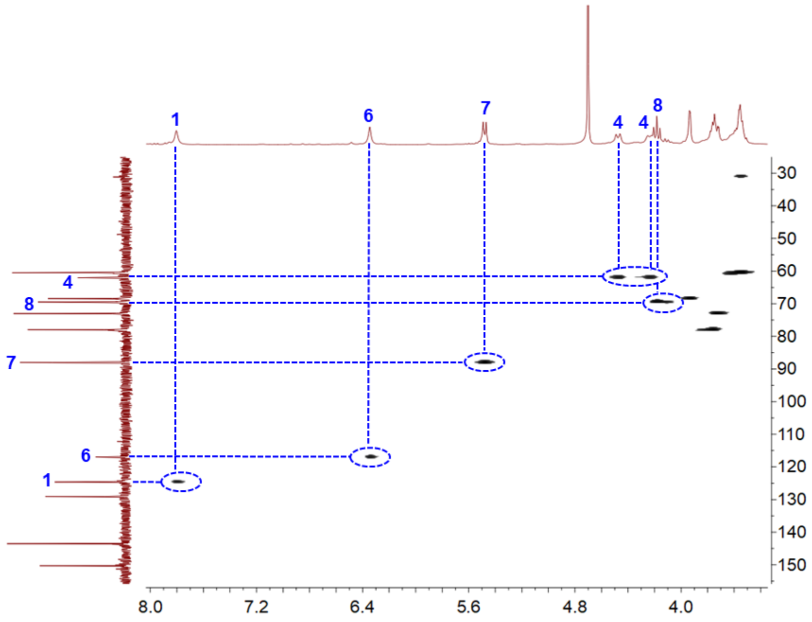

Figure S17. HSQC spectrum (400 MHz, D_2O, 298 K) of ($R_{p\text{-}D}$)-**GP5**.

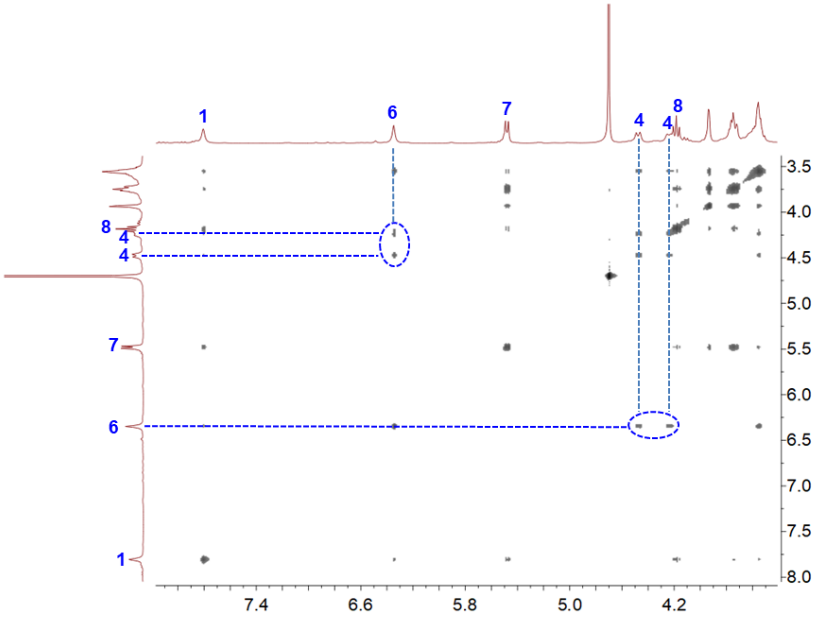

Figure S18. NOESY spectrum (400 MHz, D_2O, 298 K) of ($R_{p\text{-}D}$)-**GP5**.

3. Synthesis of control molecule

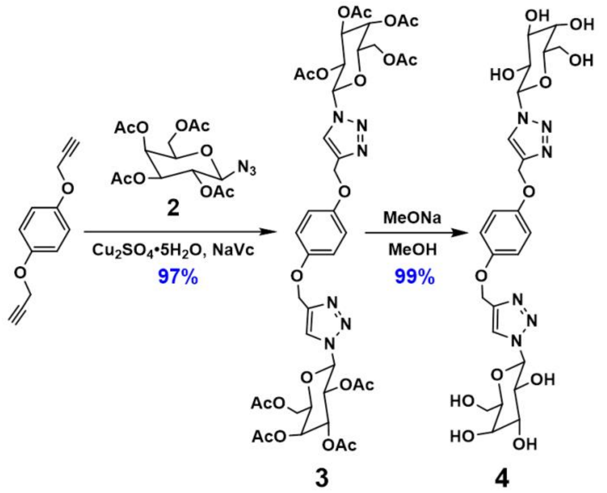

Scheme S2. Synthesis route of control molecule compound **4**.

Synthesis of compound 3

1,4-Bis(prop-2-yn-1-yloxy)benzene (0.04 g, 0.2 mmol), copper sulfate pentahydrate (0.03 g, 0.12 mmol) and sodium ascorbate (0.08 g, 0.57 mmol) were added to a solution of compound **2** (0.5 g, 1.34 mmol) in dichloromethane (10 mL). Then 10 mL H_2O was added. The mixture was stirred under Argon atmosphere in room temperature for 24 h. The reaction mixture was diluted with dichloromethane (10 mL) and washed with water (30 mL). The organic phase was dried over magnesium sulfate and filtered. The solvent was removed and the crud product was purified by silica gel chromatography (dichloromethane/methanol = 40:1, *v/v*) to afford compound **3** (0.18 g, 0.19 mmol, 97%) as a white solid. ^1H NMR (400 MHz, $CDCl_3$, 298 K) δ (ppm) = 7.92 (s, 2H),

6.94 (s, 4H), 5.87 (d, J = 9.2 Hz, 2H), 5.60-5.55 (m, 4H), 5.30-5.23 (m, 2H), 5.17 (s, 4H), 4.25-4.12 (m, 6H), 2.23 (s, 6H), 2.05 (s, 6H), 2.01(s, 6H), 1.88 (s, 6H).

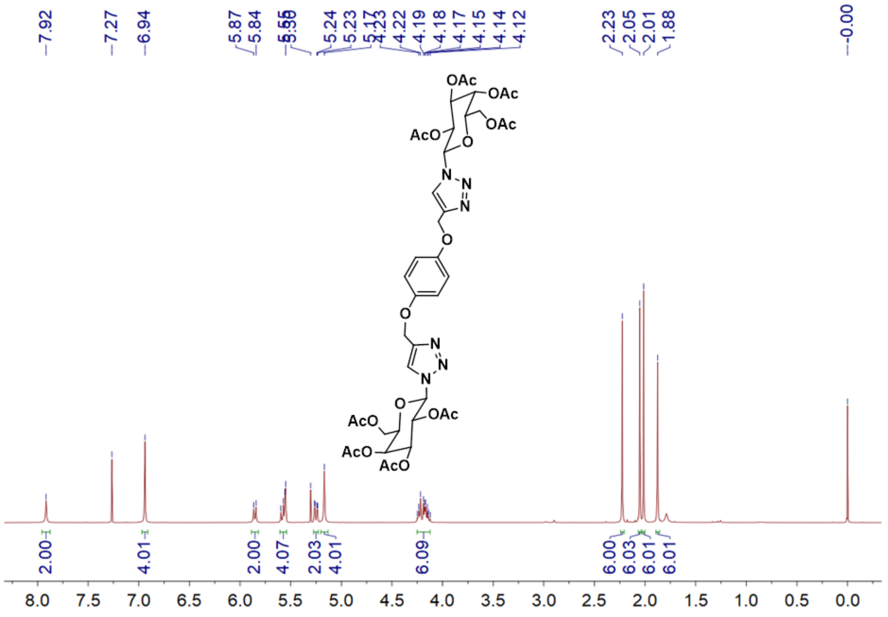

Figure S19. ^{1}H NMR spectrum (400 MHz, $CDCl_3$, 298 K) of compound **3**.

Synthesis of compound 4

Compound **3** (0.18 g, 0.19 mmol) was dissolved in a sodium methoxide solution (35 mL, 0.15 M) in a clean dry round bottom flask. The solution was stirred for 12 h. The precipitate was filtered under suction and washed with CH_3OH (5 × 20 mL) to give compound **4** (0.12 g, 0.19 mmol, 99%) as a white solid. ^{1}H NMR (400 MHz, D_2O, 298 K) δ (ppm) = 8.32 (s, 2H), 7.03 (s, 4H), 5.70 (d, J = 9.2 Hz, 2H), 5.25 (s, 4H), 4.20 (t, J = 9.2 Hz, 2H), 4.08 (d, J = 3.2 Hz, 2H), 3.99 (t, J = 6.2 Hz, 2H), 3.87 (dd, J = 9.8, 3.2 Hz, 2H), 3.78 (d, J = 6.4 Hz, 4H).

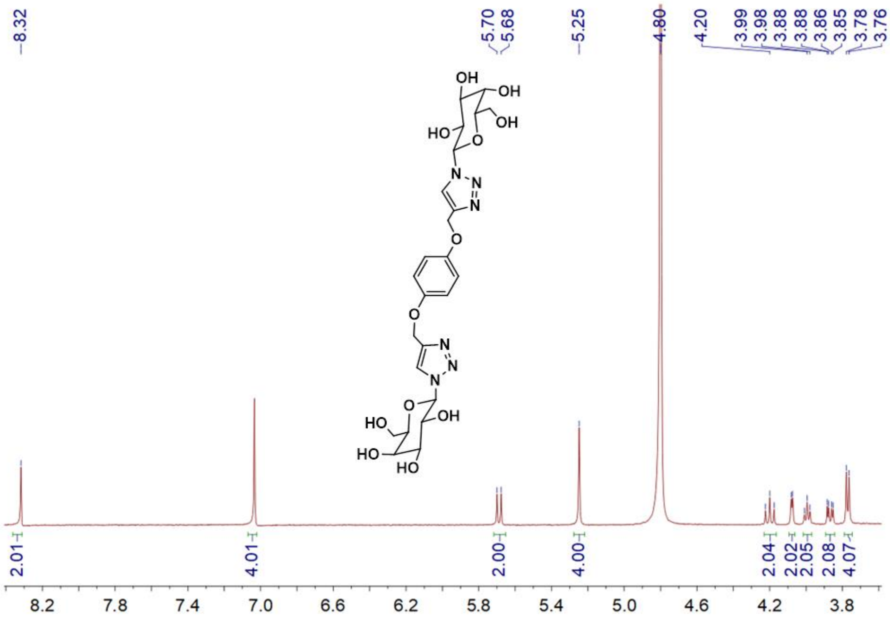

Figure S20. ^{1}H NMR spectrum (400 MHz, D_2O, 298 K) of compound **4**.

4. ¹H NMR spectra of ($S_{p\text{-}D}$)-AP5, ($R_{p\text{-}D}$)-AP5, and AP5

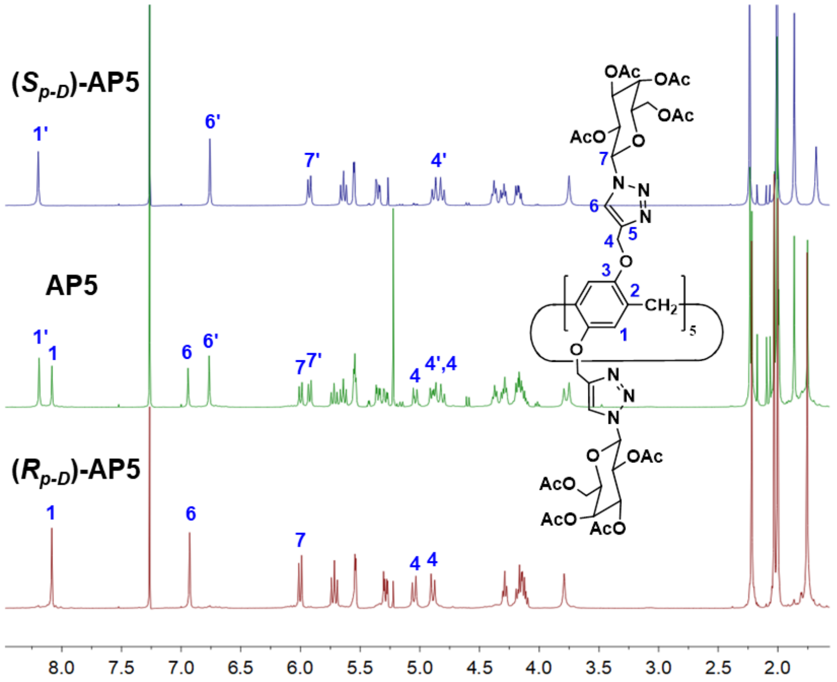

Figure S21. ¹H NMR spectra (400 MHz, $CDCl_3$, 298 K) of ($S_{p\text{-}D}$)-**AP5**, ($R_{p\text{-}D}$)-**AP5** and **AP5**.

5. ¹H NMR spectra of ($S_{p\text{-}D}$)-GP5 and ($R_{p\text{-}D}$)-GP5

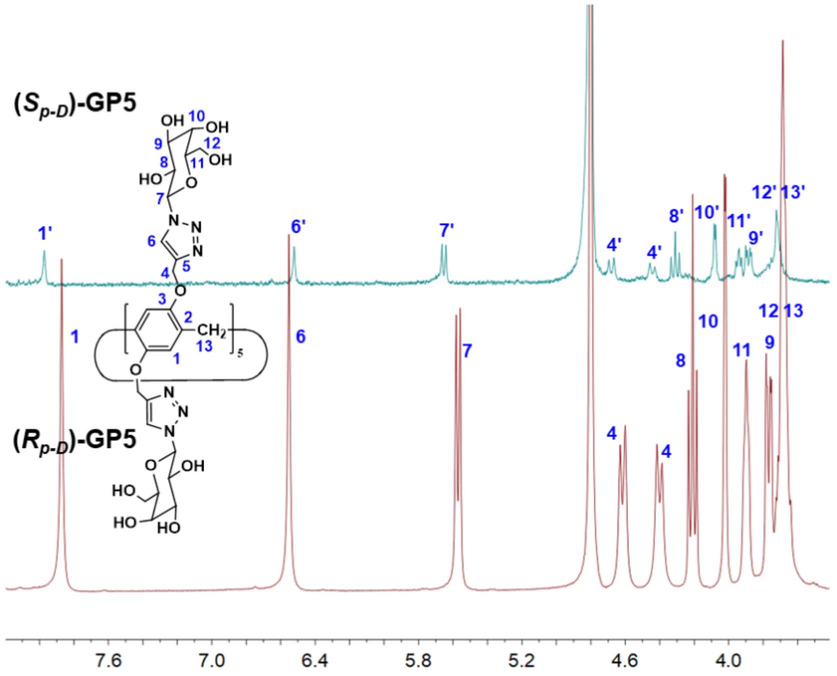

Figure S22. ¹H NMR spectra (400 MHz, D_2O, 298 K) of ($S_{p\text{-}D}$)-**GP5** and ($R_{p\text{-}D}$)-**GP5**.

6. Calculated CD spectra of ($S_{p\text{-}D}$)-GP5 and ($R_{p\text{-}D}$)-GP5

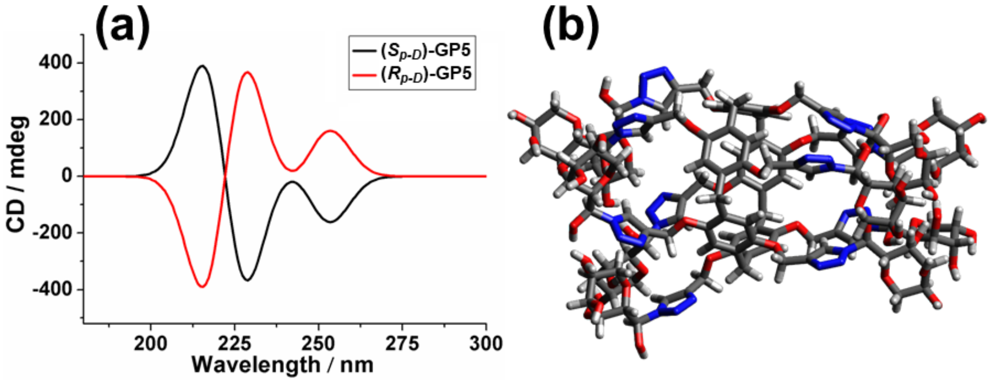

Figure S23. (a) Calculated CD spectra of ($S_{p\text{-}D}$)-**GP5** and ($R_{p\text{-}D}$)-**GP5** by using DFT method wb97xd/def2svp functional and basis set. (b) Optimized structure of **GP5**.

7. Dynamic CD spectra of ($S_{p\text{-}D}$)-GP5 and ($R_{p\text{-}D}$)-GP5

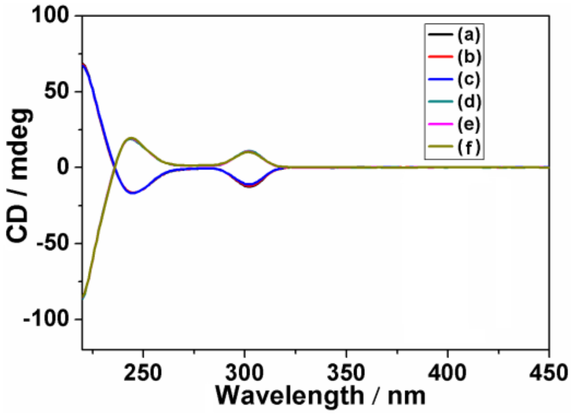

Figure S24. Dynamic CD spectra of ($S_{p\text{-}D}$)-**GP5** ((a), (b), and (c) 8 μM in H_2O) and ($R_{p\text{-}D}$)-**GP5** ((d), (e), and (f) 8 μM in H_2O) at 298, 313 and 323 K, respectively.

8. Enlarge TEM images of nanoparticles

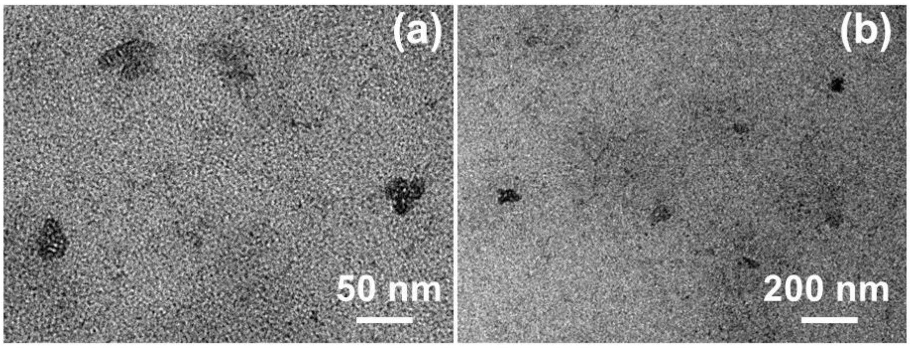

Figure S25. Enlarge TEM images of (a) ($S_{p\text{-}D}$)-nanoparticles and (b) ($R_{p\text{-}D}$)-nanoparticles.

9. Zeta potential of nanoparticles

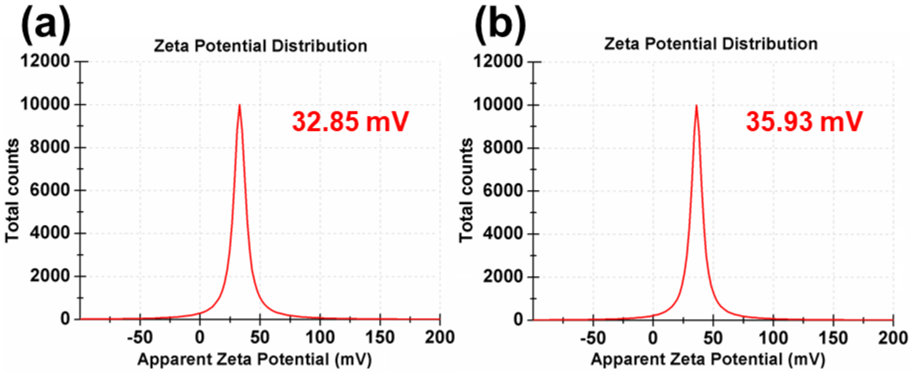

Figure S26. Zeta potential of nanoparticles: (a) ($S_{p\text{-}D}$)-nanoparticles and (b) ($R_{p\text{-}D}$)-nanoparticles.

10. References

S1. Wu, X., Zhang, Y., Lu, Y., Pang, S., Yang, K., Tian, Z., Pei, Y., Qu, Y., Wang, F., and Pei, Z. (2017). Synergistic and targeted drug delivery based on nano-CeO_2 capped with galactose functionalized pillar[5]arene via host-guest interactions. *J. Mater. Chem. B.* 5, 3483-3487. doi: 10.1039/c7tb00752c

S2. Yu, G., Ma, Y., Han, C., Yao, Y., Tang, G., Mao, Z., Gao, C., and Huang, F. (2013). A sugar-functionalized amphiphilic pillar[5]arene: synthesis, self-Assembly in water, and application in bacterial cell agglutination. *J. Am. Chem. Soc.* 135, 10310-10313. doi: 10.1021/ja405237q
